# Intussusception of Meckel’s Diverticulum: A Case Report

**DOI:** 10.7759/cureus.76513

**Published:** 2024-12-28

**Authors:** Saskia Craine, Shantam Agarwal, Zaid Al-Hamid

**Affiliations:** 1 Obstetrics and Gynaecology, Blackpool Teaching Hospitals, Blackpool, GBR; 2 Surgery, Blackpool Teaching Hospitals, Blackpool, GBR; 3 Colorectal Surgery, Blackpool Teaching Hospitals, Blackpool, GBR

**Keywords:** computed tomography scan, intussusception, meckel´s diverticulum, side-to-side anastamosis, ulcerative colitis

## Abstract

Meckel’s diverticulum (MD) is a common congenital anomaly of the gastrointestinal tract, present in approximately 2% of the population. While typically asymptomatic, MD can lead to complications such as obstruction and intussusception. Here, we present a case report of a man presenting with abdominal pain with an incidental finding of MD complicated by intussusception and our management approach.

A 39-year-old man with a history of ulcerative colitis (UC) presented with sudden severe right iliac fossa pain, vomiting, and a background of intermittent dull abdominal pain over three months. Examination revealed a tender abdominal mass in the right iliac fossa.

Despite his UC history, imaging revealed ileo-ileal intussusception with an inverted MD serving as the lead point, a rare finding in adults. The patient underwent emergency surgery, including resection of the intussuscepted bowel segment and side-to-side anastomosis. Histopathology confirmed ulceration and granulation tissue within the MD. The patient’s postoperative course was uneventful, and he was discharged with follow-up care.

This case underscores the diagnostic challenge posed by intussusception in adults, especially when overlapping symptoms suggest more common conditions like a UC flare-up. It highlights the importance of considering less frequent diagnoses, such as MD-induced intussusception, in acute abdominal presentations, and reinforces the utility of CT imaging in identifying atypical causes of bowel obstruction. The case also emphasises the need for surgical intervention in symptomatic MD cases to prevent complications.

## Introduction

Meckel’s diverticulum (MD) is the most common congenital gastrointestinal tract anomaly, with a presence of ~2% in the population [1,2]. Its pathophysiological process involves incomplete closure of the omphalomesenteric (vitelline) duct, forming a diverticulum at the antimesenteric border of the ileum. Most cases are asymptomatic; however, sometimes, there may be clinical symptoms, for example, with inflammation generating diverticulitis [2]. MD can cause other complications, including haemorrhage, intussusception, intestinal obstruction, perforation and rarely fistula, and tumours. Treatment usually involves surgical resection of the diverticulum.

## Case presentation

A 39-year-old male presented to the emergency department (ED) complaining of sudden onset, severe right iliac fossa pain and vomiting on a background of intermittent, dull abdominal pain over the last three months. The patient was opening his bowels regularly with no blood or mucus. He was diagnosed with ulcerative colitis (UC) 11 years ago and is currently on regular mesalazine and enema therapy. He attributed his symptoms to constipation or a possible flare-up of UC. He regularly consumes between 13 and 16 beers a day and smokes marijuana weekly. 

On examination, he was apyrexic and haemodynamically stable. Abdominal examination revealed a tender mass in the right iliac fossa. Peritonism and guarding were absent. Bowel sounds were sluggish on auscultation.

**Figure 1:**
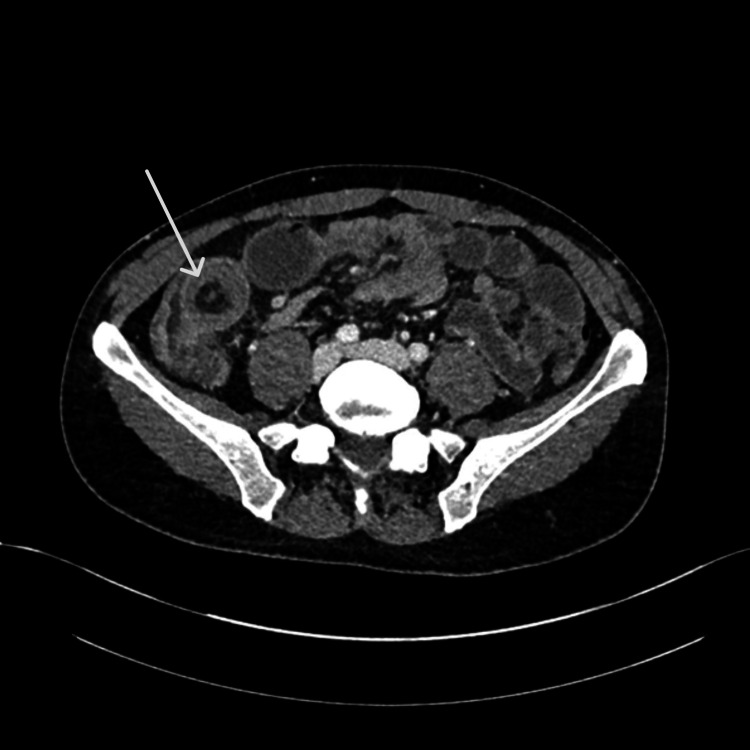
CT-AP scan of the patient, axial view: Ileo-ileal-intussusception within terminal ileum (marked with white arrow) with surrounding oedema and proximal dilatation of the small bowel.

With respect to investigations on admission, laboratory findings (Table 1) were insignificant. The patient was subsequently booked for a computed tomography (CT) scan to rule out appendicitis. He underwent a CT of the abdomen and pelvis (CT-AP), shown in Figure 1, which revealed a large segment of the small bowel was intussuscepted within the terminal ileum.

**Table 1:** Patient parameters on admission

Parameter	Patient values	Reference range
White cell count (WCC)	10.6 × 10^9^/L	4.5-11.0 × 10^9^/L
Neutrophils	9.63 ×10^9^/L	1.5-8.0 × 10^9^/L
C-reactive protein (CRP)	1.4 mg/L	8-10 mg/L
Haemoglobin (Hb)	154 g/L	138-172 g/L

The patient was subsequently listed for emergency surgery. At laparotomy, ileo-ileal intussusception with an inverted MD was noted. The diverticulum was the lead point of the intussusception. The intussuscepted small bowel segment was resected, and the remaining segments of the bowels were anastomosed via side-to-side anastomosis. 

The surgical specimen consisted of a section of a small bowel measuring 16.5 cm in length and 4 cm in diameter with a tubular outpouching of everted bowel. Histopathological examination of the outpouching demonstrated areas of ulceration with granulation tissue. The patient’s postoperative events were unremarkable, and he was discharged home four days later with a general surgery follow-up.

## Discussion

Intussusception occurs when a segment of the bowel telescopes into an adjacent bowel segment, leading to complications such as obstruction or intestinal ischaemia [1]. The condition is more common in children and presents with cramping abdominal, red currant jelly stool, and a palpable tender mass. Paediatric intussusception is usually benign, and a reduction is sufficient to treat the condition in 80% of cases. In adults, bowel intussusception is regarded as uncommon, representing 5% of all intussusception cases and 1-5% of intestinal obstruction [1,3]. 

The cause of bowel intussusception remains unidentified in 8-20% of primary or idiopathic cases, and it is more likely to occur in the small intestine [1]. The remaining cases of adult intussusception (secondary) are due to a pathological lead point, which can be intraluminal, mural, or extramural, which alters normal peristaltic activity and initiates the inversion of one segment of the bowel into the other [4]. This interferes with peristalsis and hinders the movement of intestinal contents. More critically, it can compromise the mesenteric blood flow to the affected bowel segment, resulting in bowel obstruction and inflammatory changes that can range from thickening of the bowel wall to ischaemia [4]. This interferes with peristalsis and hinders the movement of intestinal contents. More critically, it can compromise the mesenteric blood flow to the affected bowel segment, resulting in bowel obstruction and inflammatory changes that can range from the thickening of the bowel wall to ischaemia [4].

Most cases of intussusception in adults with UC are caused by a neoplastic lesion, especially colonic intussusception, in which a tumour-related lesion serves as a lead point in 75% of presentations [5]. Other potential lead points include inflammatory polyps, MD, colonic diverticulum, cytomegalovirus, strictures, or benign neoplasms. Among adults, neoplastic lead points are the most frequent cause of intussusception [4,6].

In the present case, when the laparotomy procedure was initiated, an incidental finding of an MD was found in the ileo-ileal intussuscepted segment of the small bowel. Therefore, MD is thought to be the lead point of this patient’s bowel intussusception, and not neoplasm or an inflammatory polyp, which are known to be more common causes of bowel intussusception in adults with UC [7]. 

MD is the most prevalent congenital abnormality of the small intestine, found in about 2% of the population [1,2]. Rarely can MD invert into the ileal lumen and can be the lead point for intussusception, as seen in the present case. The cause of inversion is not clearly understood; however, abnormal peristaltic movement resulting from ulceration or ectopic tissue at the base of MD may lead to its inversion [8]. Intussusception caused by MD is estimated to account for 4% of all intestinal obstruction cases related to intussusception [4]. Preoperative diagnosis can be difficult because, clinically, patients with intussusception frequently exhibit symptoms that overlap with those of other conditions, such as bowel obstruction. These symptoms include vomiting, acute abdominal pain, tenderness, and distension [4]. Chen et al. (2014) conducted a literature review on MD clinical manifestations and found 40.4% of MDs were found incidentally during surgery for other diseases, and 67% had inflammation of MD [9]. Groebli et al. (2001) conducted a similar study and found that 56% of MD diverticula were resected during laparotomy for another reason, and only 47% had symptoms caused by the diverticula [10]. Additionally, in 90% of those who had a CT, none suggested MD as a cause of symptoms [10], as seen in the present case. 

## Conclusions

This case represents a complex interplay of medical and surgical pathologies. On presentation of this patient to the ED, considering the patient’s background history of UC, a UC flare-up seemed to be the most obvious diagnosis. However, the history of sudden onset, severe abdominal pain on a background of a three-month history of dull abdominal pain, and examination findings of a distended abdomen and abdominal mass were all signs of a more suspicious pathology and were the reason for further investigating the case with a CT scan. A CT is the most useful examination for diagnosing intussusception as it provides an accurate diagnosis in most patients. Nevertheless, an incidental finding of MD during surgical resection, such as in this case, is not uncommon. This case highlights the importance of “thinking outside the box” when a patient presents to the ED with acute abdominal symptoms.
